# Good practice in mental health care for socially marginalised groups in Europe: a qualitative study of expert views in 14 countries

**DOI:** 10.1186/1471-2458-12-248

**Published:** 2012-03-28

**Authors:** Stefan Priebe, Aleksandra Matanov, Ruth Schor, Christa Straßmayr, Henrique Barros, Margaret M Barry, José Manuel Díaz-Olalla, Edina Gabor, Tim Greacen, Petra Holcnerová, Ulrike Kluge, Vincent Lorant, Jacek Moskalewicz, Aart H Schene, Gloria Macassa, Andrea Gaddini

**Affiliations:** 1 Unit for Social and Community Psychiatry, Queen Mary University of London, London, UK; 2 Ludwig Boltzmann Institute for Social Psychiatry, Vienna, Austria; 3 Department of Hygiene and Epidemiology, University of Porto Medical School, Porto, Portugal; 4 Health Promotion Research Centre, National University of Ireland Galway, Galway, Ireland; 5 Madrid Salud, Madrid, Spain; 6 National Institute for Health Development, Budapest, Hungary; 7 Laboratoire de recherche, Etablissement Public de Santé Maison Blanche, Paris, France; 8 Department of Psychiatry, 1st Faculty of Medicine, Charles University, Prague, Czech Republic; 9 Clinic for Psychiatry and Psychotherapy, Charité, University Medicine Berlin, Berlin, Germany; 10 Institute of Health and Society (IRSS), Université Catholique de Louvain, Bruxelles, Belgium; 11 Institute of Psychiatry and Neurology, Warsaw, Poland; 12 Academic Medical Center, University of Amsterdam, Amsterdam, The Netherlands; 13 Department of Public Health Sciences, Karolinska Institute, Stockholm, Sweden; 14 Laziosanità ASP - Public Health Agency, Lazio Region, Rome, Italy

**Keywords:** Marginalisation, Mental health care, Health care systems, Good practice, Autonomy

## Abstract

**Background:**

Socially marginalised groups tend to have higher rates of mental disorders than the general population and can be difficult to engage in health care. Providing mental health care for these groups represents a particular challenge, and evidence on good practice is required. This study explored the experiences and views of experts in 14 European countries regarding mental health care for six socially marginalised groups: long-term unemployed; street sex workers; homeless; refugees/asylum seekers; irregular migrants and members of the travelling communities.

**Methods:**

Two highly deprived areas were selected in the capital cities of 14 countries, and experts were interviewed for each of the six marginalised groups. Semi-structured interviews with case vignettes were conducted to explore experiences of good practice and analysed using thematic analysis.

**Results:**

In a total of 154 interviews, four components of good practice were identified across all six groups: a) establishing outreach programmes to identify and engage with individuals with mental disorders; b) facilitating access to services that provide different aspects of health care, including mental health care, and thus reducing the need for further referrals; c) strengthening the collaboration and co-ordination between different services; and d) disseminating information on services both to marginalised groups and to practitioners in the area.

**Conclusions:**

Experts across Europe hold similar views on what constitutes good practice in mental health care for marginalised groups. Care may be improved through better service organisation, coordination and information.

## Background

The concept of social marginalisation refers to ‘social isolation and/or inability to be able to fully participate in the standards and way of life of society’ [1]. It is linked to social exclusion [2,3], and is frequently considered a consequence of economic marginalisation [4]. Various studies show a higher prevalence of psychiatric disorders in marginalised groups than in the age-matched general population [5-7]. Providing mental health care for people from these groups represents a particular challenge.

Marginalised groups can face significant administrative and financial obstacles in accessing health services [8-11] and be neglected in the distribution of health resources [12]. Services providing mental health care can struggle to reach people with mental disorders in these groups and engage them in care. Compounding these various difficulties, there is limited systematic research evidence to guide service provision for these groups.

In its health strategy, the European Commission promotes the values of universality, access to good quality care, equity and solidarity and is committed to reducing health inequalities [13,14]. In 2011 the European Parliament adopted a resolution on “Reducing health inequalities in the EU” in which Member States are urged to focus on the needs of vulnerable groups [15]. The resolution recognises that the risks of health inequalities are magnified by the combination of poverty and other vulnerabilities, and that they can be related to problems of access to health care. The EC Green Paper on mental health [16] emphasises support for vulnerable groups as one of the key aspects of mental health promotion.

Against this background, this study aimed to identify components of good practice in the provision of mental health care across six groups that are widely considered as socially marginalised [17]: long-term unemployed; street sex workers; homeless; refugees/asylum seekers; irregular migrants and members of the travelling communities.

We collected data in 14 European countries with a consistent methodology to arrive at findings that are not limited to a specific group or context. Assuming that social marginalisation is more frequent and prominent in deprived urban areas, we focused on two highly deprived areas in each of the capital cities. Interviews were conducted with experts for each group in each area to explore components of good practice across all groups.

## Methods

The study was part of the PROMO project (‘Best Practice In Promoting Mental Health In Socially Marginalized People In Europe’) which was funded by the European Commission (DG Sanco) and conducted from 2007 to 2010 [18]. The project was carried out in 14 countries: Austria, Belgium, Czech Republic, France, Germany, Hungary, Ireland, Italy, Netherlands, Poland, Portugal, Spain, Sweden, and the United Kingdom.

### Definition of marginalised groups

The definition of long-term unemployed was based on the EUROSTAT definition: a person of the national working age, who has been out of employment for twelve months or longer [19]. The definition of sex workers focused on individuals selling sexual services outdoors, due to some evidence of greater vulnerability of this group [20]. The definition of homelessness encompassed two categories of the ETHOS typology: rooflessness (sleeping rough or in emergency accommodation) and houselessness (sleeping in hostels or other temporary accommodation) [21]. Asylum seekers and refugees were defined in relation to the 1951 UN Convention Relating to the Status of Refugees [22]. An asylum seeker was defined as a person who is seeking international protection by applying for refugee status as defined by the 1951 UN Convention, but whose claim has not yet been decided by the relevant authorities. A refugee was defined as a person who has been granted such a status. Irregular migrants were defined as those who are not in possession of a legal residency permit in the host country, which includes failed asylum seekers. Travelling communities were defined as any communities that are committed to a nomadic or travelling lifestyle and/or see travelling as an important part of their cultural identity. This definition also included those who are settled but face marginalisation because of associations with travelling lifestyle traditions.

### Selection of deprived areas

A total of 28 highly deprived geographical areas, two in each participating capital city, were identified using local indices of public health and social deprivation. The population size of each area was intended to be between 80,000 and 150,000 inhabitants, with some flexibility to accommodate different local contexts. If chosen areas were too small, several areas were combined to achieve the target size. The selected areas were: Vienna: District 16 and District 20; Brussels: Schaerbeek & St Josse and Molenbeek; Prague: Prague 3 & 7 and Prague 8; Paris: Secteur Flandre in the 19^th^ arrondissment of Paris and La Courneuve & Aubervillers in Seine Saint Denis; Berlin: Wedding and Kreuzberg; Budapest: District 8 and District 7 & 9; Rome: District 7 and District 15; Dublin: Dublin North Central and Dublin West; Amsterdam: Bos en Lommer & De Baarsjes & Geuzenveld-Slotermeer and Amsterdam Zuid Oost; Warsaw: Praga Polnoc and Wola; Lisbon: Marvila & Santa Maria dos Oliváis and a group of smaller areas (Anjos, Castelo, Encarnação, Graça, Madalena, Mercês, Pena, Penha de França, Santa Catarina, Santa Engrácia, Santa Justa, Santiago, Santo Estêvão, Santos-o-Velho, São Cristóvão e São Lourenço, São José, São Miguel, São Nicolau, São Paulo, São Vicente de Fora, Sé, Socorro); Madrid: Villaverde and Centro; Stockholm: Rinkeby-Kysta & Spånga-Tensta & Skarpnäk and Sodermalm; London: Hackney and Tower Hamlets.

### Recruitment of experts

In each of the 28 deprived areas, we set out to find a practitioner with expertise based on experience in providing mental health care for each of the six marginalised groups. Using a wide understanding of mental health care, we attempted to contact all services providing some form of mental health care in the given area to identify suitable interviewees. We included interviewees with a professional background in mental health care, general health care or social care. The criteria for inclusion were good knowledge of local service provision and experience of providing or facilitating access to mental health care for people from the six groups. If such an expert could not be found in the area, they were recruited from other areas in the same city. In some cases the same person had expertise for several marginalised groups and was interviewed for more than one group. Potential participants were employees of a wide range of services in the not-for-profit and state sector. They were contacted by the researchers via telephone or email. A detailed description of the study was provided to all potential participants, anonymity was assured and informed consent obtained. All interviews were carried out face-to-face and audio-taped by the researchers in the participating centres. The occupational background of the interviewees and the nature of their professional involvement with socially marginalised groups were documented.

### Interviews

A semi-structured interview schedule was developed in an iterative process involving all partners and translated into the languages of all participating countries. Based on one pilot interview in each country the schedule was modified and finalised. Each schedule consisted of two case vignettes, adapted for each of the six marginalised groups, and four general questions on the quality of mental health care in the given area for that group. The vignettes described two patients with different mental health problems, one exhibiting symptoms of psychotic and the other depressive disorder, with each having different attitudes towards seeking care. The experts were asked questions about their pathways into care for these patients, including the ways of obtaining relevant information, services that were likely to respond initially to their needs, and further treatment options. They were also asked about barriers that patients may encounter and ways to overcome them. The same twelve vignettes (two for each target group) were used across all of the capitals to ensure consistency. The four general questions addressed the quality of mental health care provided to the specific group in the a given area in terms of strengths, weaknesses, co-ordination of services and suggestions for improvement.

Ethical approval was not required for the study in the participating countries, as no patient data was collected.

### Data analysis

All interviews were transcribed by the researchers in the participating centres, ensuring the removal of any identifying information so as to maintain anonymity. For the overall analysis, the relevant material was translated into English by the same researchers. The coordinating centre examined the translated transcripts and sought any necessary clarifications from the respective centres.

The data were analysed using thematic analysis [23]. This was carried out by the members of the multi-disciplinary core research team in the coordinating centre, in regular consultation with the wider PROMO group. In the first stage of the analysis, the data from the initial transcripts for each of the PROMO groups was coded line-by-line. The resulting coding frame was used to code the rest of the transcripts. The researchers from different participating centres were involved in the initial coding and the development of the coding frame. The second stage of the analysis involved the extraction of categories from the codes and their subsequent refinement and grouping into conceptual themes [24,25]. Frequency counts of themes and the corresponding categories were also recorded. The analysis was first performed for each of the six groups and then synthesised stepwise into overarching themes. These themes represented commonalities in the components of good practice across all groups and were based on aspects that were raised in more than one country. Themes specific to only one or two of the six groups or the national context of only one country, e.g. specific aspects of the national funding system for services, were not included. The emerging themes were regularly revised in the multi-disciplinary study team at the co-ordinating centre, discussed in the wider international project group, and further refined and specified with ongoing checks against the material. MAXqda (v.10) software was used for the analysis.

The professional background of the core research team in the coordinating centre included social psychiatry, public health, psychology, social sciences, social work and policy. Their expertise comprised both academic and clinical experience. Three researchers were involved in management of mental health services and governance of a Mental Health Trust (an organisation providing mental health services in the English National Health Service). The wider international PROMO research team was also multidisciplinary with a range of different qualifications.

## Results

### Participants

We identified and contacted 162 participants in fourteen countries. Out of the two refused to participate: one in Germany and one in the Czech Republic. In total, assessments were conducted with 160 experts, with data from 154 being included in the analysis (Austria: 11; Belgium: 13; Czech Republic: 10; France: 12; Hungary: 10; Germany: 13; Ireland: 12; Italy: 12; Netherlands: 12; Poland: 12; Portugal: 10; Spain: 10; Sweden: 6; UK: 11). The six excluded interviews were omitted due to poor quality of the recording, missing or duplicated information. In four cases information provided for the assessment was insufficient and was complemented by an additional expert.

With respect to the professional background of the participants, we interviewed 20 psychiatrists, 26 psychologists/psychotherapists, 55 social workers, 3 occupational therapists, 9 nurses, 12 medical doctors, 13 community workers, 2 lawyers and 14 participants with social science/policy background.

The participants were currently employed in either mental health care positions (n = 53), social care positions (n = 79), general health care positions (n = 19) or academic positions (n = 3). 18 were managers/coordinators of mental health and 32 of social care services.

We analysed 25 assessments for the long-term unemployed, 26 for street sex workers, 28 for the homeless, 27 for asylum seekers and refugees, 25 for irregular migrants and 23 for travelling communities. In the case of the long-term unemployed, irregular migrants and travelling communities, in three capitals a single expert provided the assessments for both areas. For street sex workers, a single expert was interviewed in two capitals.For travelling communities, expert views were obtained in all countries other than Spain. For the other five marginalised groups, expert opinions were obtained in all 14 countries.

### Components of good practice

The interviews varied in length, detail and the extent to which experiences and opinions concerning good practice were explained. In many interviews there was a tendency to emphasise barriers to providing good care and weaknesses of the given local care system rather than positive aspects and clear ideas for what might constitute good practice.

The analysis identified 13 themes that were then grouped into four components of good practice: outreach programmes, facilitating access to general health services, collaboration and co-ordination of services, and information.

Table 1 shows the number of areas and countries for which experts raised each theme (since there usually expert views for two areas and each group in each country).

**Table 1:** Total number of geographical areas and countries for which each theme was raised

**PROMO groups**	**Long-term unemployed**^**1**^	**Street sex workers**^**2**^	**Homeless population**^**3**^	**Refugees/ Asylum seekers**^**4**^	**Irregular migrants**^**5**^	**Travelling communities**^**6**^
**Outreach Programmes**						
Provision of outreach services	10/6	20/11	18/11	6/5	6/5	12/9
Trust building, non-intrusive approach	6/4	17/10	19/12	11/7	9/6	15/9
Ensuring regular contact/continuity of support	6/6	14/9	11/8	4/4	-	9/7
**Facilitating access to general**						
**health services**						
Assisting patients in obtaining health coverage	5/5	11/8	12/7	14/10	17/12	11/6
Flexibility of access and referrals	5/5	11/7	6/5	6/6	7/5	11/6
Awareness training for health and social care services staff on living conditions and the needs of a specific group	-	12/7	7/6	8/7	7/6	16/11
Mental health training for health and social care staff in frontline services	8/6	2/2	5/4	2/2	-	-
Provision of teams/professionals with specialised knowledge & skills	2/1	13/8	12/7	4/4	3/3	8/5
**Collaboration and co-ordination of services**						
Good collaboration between mental health, social care services and services specific to the group	18/12	20/12	19/10	17/10	16/12	19/13
Developing integrated services/protocols	7/7	7/5	6/5	14/9	4/3	2/2
Exchange of expertise between different types of professionals	7/7	7/5	2/2	6/6	2/2	4/3
**Information**						
Provision of information on mental health services and care available	6/5	14/8	2/2	10/7	8/6	7/5
Provision of health care and/or health entitlement education	4/4	5/4	2/2	15/11	12/8	7/5

^1 2 3 4 5^out of 28 areas and 14 countries; ^6^out of 26 areas and 13 countries.

### Outreach programmes

Mainstream mental health services commonly expect people with mental disorders to be active in seeking treatment. Experts saw this as unrealistic for people in socially marginalised groups. Some of them such as irregular migrants or those without appropriate insurance cover may not be entitled to use services and therefore cannot easily access mainstream care. Even when people in marginalised groups are fully entitled to care, their life styles may not be in line with the requirements of services (e.g. using services within office hours, arranging and keeping appointments). Furthermore, they may have insufficient knowledge or skills to access mainstream services, feel ashamed to go to services and talk about their background, or lack trust in services and staff. For all of these reasons, experts saw a need for establishing outreach programmes that seek to initiate contact with marginalised groups, establish a relationship of trust, identify people with mental disorders and assist them in accessing care. It was proposed that care can be provided in the existing welfare and community organisations that people from marginalised groups already use and trust.

“We need to take more of an outreach approach in terms of care. The old fantasy of people needing to ask in order for anything to happen is obsolete. It is time for local community mental health centres to authorise outreach…..”

Paris, mental health professional for the homeless, Id. 44

Experts noted that professionals involved in outreach programmes are able to meet people in their own environment and assess their needs more accurately. They are also able to identify barriers to accessing services and find ways of surmounting them.

“We try to bring services to the women and it does make a difference; it shows that you care enough…We see how their life is. We go out at night and see how it works – we try to get our collaborators from other services to come out with us to see how it is to be on the street.”

London, social care professional for street sex workers, Id. 178

The process of building trust with people from marginalised groups was described as the essential characteristic of outreach programmes. This is achieved through regular contact with the groups, which helps potential patients to overcome mistrust and accept care.

“…something very fundamentally important about sex workers has to be said, something that applies to every marginalised group: it needs time, sometimes a long time for the women to have trust. So if someone does street outreach on one occasion and then only turns up a year later, the women will not trust them. So it needs continuous work.”

Vienna, social care professional for street sex workers, Id. 10

“We try to get in contact with homeless people. We try to listen to them. We “adopt” their reference system and their outlook on life. In this way we try to understand how to meet their needs, we act as facilitators…We try to understand why people don’t collaborate. In case of so called “non-collaborative” people, we believe that we cannot just call them “non-collaborative” – it is a way to test us. Thanks to our experience we try to understand their defence mechanisms in order to help them”.

Rome, mental health professional for the homeless, Id. 94

Outreach services were seen as important for providing care for “invisible” groups such as irregular migrants who may face legal difficulties.

“…irregular immigrants are often afraid to ask for help in organisations, they use only those recommended by other immigrants. They find us thanks to recommendation by others. And they always need to be reassured that we will not ask them for documents.”

Warsaw, social care professional for irregular migrants, Id. 121

“…what happens in the case of homeless immigrants…. it is that only an outreach service will “catch them.” We can either go and reach them where they are, or otherwise they will not come to the national health service.”

Lisbon, social care professional for refugees/asylum seekers, Id. 132

The experts emphasised that many members of marginalised groups have a history of negative experiences with various statutory services and authorities. Consequently, non-intrusive approaches and a respect for autonomy were seen as essential to working with them.

“It often requires good clinical and communication skills to establish a working relation with a client such as this. Often it is advisable not to discuss any psychiatric matters during the first one or two sessions in order to gain some trust…; even if contact is made between a client and a mental health professional, it may not result in actual treatment because some clients refuse further cooperation if they are not approached in the right way or due to mistrust or previous negative experiences with psychiatric treatment. Mental health care professionals should be aware that it is often essential to invest time in establishing a good relationship with a patient before treatment can be started”

Amsterdam, mental health professional for the homeless, Id. 105

“They are very suspicious. It is very difficult for them if they have been sleeping rough. You need to find out what their previous experiences have been and adopting a gentle, gentle approach to helping the client overcome some of the barriers.”

Dublin, mental health professional for the homeless, Id. 177

Where outreach services already exist, they should include mental health expertise. These services may or may not provide a substantial part of mental health care themselves.

“Street outreach for first stage clients in collaboration with statutory mental health teams is useful. Where this is not available it is harder to get access to other services.”

London, mental health professional for the homeless, Id. 170

Providing mental health care within welfare and community organisations that people with mental health problems already use and trust was seen as an effective way to reduce stigma and fear associated with mental illness, as potential patients are approached in familiar and non-threatening environment.

“Mental health services make sense, and psychological support in particular, in places where there are other services that people usually attend, for example to get their documentation.”

Lisbon, social care professional for refugees/asylum seekers, Id. 131

“And of course that’s always a good thing because people are in their familiar environment. It always creates trust when we say that they can meet the person from X (a psychiatric service) here in our office. They do not feel “Oh my god, now I have to go to the local authorities, and they are going to keep me there and I’ll be taken away.” In mentally ill persons, that fear is often at the back of their mind…”

Berlin, social care professional for the long-term unemployed, Id. 74

Outreach work was also reported as a way to build bridges with communities that lack trust in authorities due to prejudices and/or different cultural values, and explanatory models that are not well understood or appreciated in mainstream services.

“I convinced the hospital authorities to dispatch a nurse, paid by the hospital, to work with the Tsiganes. She spends half her time doing fieldwork with the families and at the hospital so that if a Tsigane patient is hospitalised, she can act as a go-between. We really need to do everything we can to bring the two cultures closer”.

Paris, social care professional for travelling communities, Id. 38

### Facilitating access to general health services

If outreach programmes are the first point of contact, they may have to refer patients to other services with a wider or more specialised expertise. Patients need help in overcoming administrative barriers to benefiting from health care services that address a wide range of health problems (as various primary care services do). This ensures a multidisciplinary approach and also avoids an unnecessary split between physical and psychological problems. Providing physical and mental health care in one service can also increase the acceptability of treatment for mental disorders.

People from socially marginalised groups are frequently found to be “outside” health care systems. For some groups this is due to limited health care entitlement, however, even those with full entitlement often do not have the necessary documents. Obtaining health insurance is frequently linked to complex requirements and time-consuming procedures that patients from marginalised groups may struggle to follow.

“The health care card is only available if certain conditions are satisfied: social security number, identification document, proof of address, tax payer number. Unfortunately, this depends very much on the person they run into: with hospitals and primary health care administrative staff, if there are some that make it easy, there are others that create barriers that shouldn’t even exist ”

Lisbon, social care professional for refugees/asylum seekers, Id. 131

Not having apropriate insurance limits access to health care. This applies in particular to patients requiring long-term treatment, as is often the case for mental illness. The experts emphasised the need to speed up and simplify the process of obtaining insurance and to provide assistance to patients when necessary.

“We managed to work out efficient solutions in terms of medical help for the homeless in Wola. We …have managed to convince the directors of all the hospitals in our district to employ social workers in their facilities. Thanks to this solution, when a homeless person comes to them, they can immediately start the procedure (of obtaining insurance) – it’s also beneficial for the hospitals themselves, as it gives them certainty they will receive payment for the services delivered to the homeless. Our aim was to shorten the official process for insuring homeless people, so they can make use of the full range of services.”

Warsaw, social care professional for the homeless, Id. 116

People from marginalised groups frequently find it difficult to follow complex administrative procedures linked to admission and referals. Making these procedures as flexible as possible was seen as an important aspect of good practice.

“Making access to services as bureaucracy- free as possible …..really minimum paperwork, where they are able to do paperwork in small chunks.”

London, social care professional for street sex workers, Id. 178

“… in our department, we adapt our environment to these patients; in other words we know how difficult it is for them to organise their time, the complicated relationship they may have with time and we try to take that into consideration by being flexible.”

Paris, mental health professional for irregular migrants, Id. 47

Training health care staff about the specific circumstances and needs of different marginalised groups was also seen as essential for delivery of appropriate and effective care.

“…what I consider very important is a vocational training for health care staff. Staff are giving asylum seekers well-intentioned advice, which is completely ineffective because they have no idea what these people need and in what conditions these people are living. This would save costs and people’s health would improve faster and they could easier integrate more easily once they obtain asylum.”

Vienna, mental health professional for refugees/asylum seekers, Id. 6

Encouraging patients to use mainstream services was seen as important in preventing isolation and fostering sense of agency:

“…people from the homeless centre should also use the nearest public clinic. It’s important for these people - we mustn't isolate them by offering them separate services. What we need to do is to include the homeless persons in the mainstream care services but at the same time we need to maintain an individual approach and create several special services for the difficult cases - to reach everybody in need.”

"Warsaw, social care professional for the homeless, Id. 116"

### Collaboration and co-ordination of services

In practically all areas studied different generic and specialised services were involved in providing mental health care for different marginalised groups. Experts reported that these services often work independently of each other. They stated that there was too little direct collaboration and information sharing, and an absence of overall co-ordination of services for such groups.

“Services have to acknowledge the holistic issues, and link with other organisations dealing with those clients. Often nobody knows the whole picture. It’s exhausting and depressing to tell your story over and over again to different agencies – services need to be set up with more joint working.”

London, health professional for travelling communities, Id. 176

This may be linked to a general fragmentation of the health and social care system, but was seen as particularly problematic for marginalised groups since they often present with multiple needs, and require smoother collaboration between services to benefit from the whole system. Also, good collaboration and coordination were regarded as essential to optimise the use of often restricted resources, and to ensure that patients from these groups are not neglected because no service takes responsibility.

*“There really has to be a network. Otherwise, each party will just pass the buck. Social services say it’s psychiatric and will not provide care, psychiatry says it’s a homeless person and they do not handle that and while we’re busy going back and forth, nothing gets done*…*We have to meet regularly, agree on how to articulate patient care, on who does what. This is true for all patients but it is even truer for patients with serious difficulties on the social level”*

Paris, mental health professional for the homeless, Id. 45

Collaboration with group-specific services and community organisations was seen as valuable due to the knowledge of the living circumstances and needs of specific groups that workers in these services had and the fact they were already trusted by potential patients.

“These cases were often identified by the immigrant associations…. definitely, the great advantage that the immigrant associations have in this field was precisely their proximity with the population they represented.”

Lisbon, social care professional for refugees/asylum seekers, Id. 132

Exchange of expertise between different services through sharing posts and mutual training was identified as an important aspect of collaboration which can lead to a greater shared understanding between the different care sectors and better ways of working together:

“There are also exchanges of workers with these outpatient mental health services. For example, a psychologist from outpatient mental health service came for several months in our team to discover the "universe" of homeless people, and we learned from her about mental health.”

Brussels, social care professional for the homeless, Id. 23

“Voluntary sector staff benefit from contact with mental health teams. New workers can learn from the statutory worker and this is good for the client. You learn about pressures on the statutory system if you are in the voluntary sector. They (statutory sector) are suffering from the same difficulties as you are. Services which we think exist don’t exist. Knowing this may help us to do things better and to understand the options for these clients moving on”

London, social care professional for the homeless, Id. 170

“We could actually give more by sharing our knowhow than by trying to help every single person who comes to us. Support and encourage other dedicated centres, so that people are no longer scared to deal with this population.”

Paris, mental health professional for refugees/asylum seekers, Id. 42

**“**We have excellent collaboration with the social workers. Their competence allows us to understand better where to place the patient. Their understanding of the patient’s needs help us to find the solutions**.”**

Rome, mental health care professional for the long-term unemployed, Id.99

### Information

Linked to the above issue of collaboration and co-ordination was the often insufficient information about services and care provision. Professionals from statutory and non-statutory social care and community organisations often found it difficult to obtain information about available mental health services and to navigate complex referral systems. Sufficiently comprehensive information on services in a given area was not seen as difficult to put together and make available to all relevant organisations in the area, including community groups and other agencies that may be in a position to provide advice to people from marginalised groups.

“All institutions and organisations involved in activities in this field [should] know their own and others’ responsibilities and available services. Also some kind of a … recommended solutions and procedures guide… a guide for social workers, employees of NGOs and others…”

Warsaw, social care professional for unemployed, Id. 117

On the other hand, mental health professionals from statutory services emphasised the need for information on other services and agencies that provide support to marginalised groups, and on problems specific to certain marginalised groups, ie immigration procedures or health entitlement regulations.

*“Our organisation is busy working on a guide for the mental health social workers which will gather information about interpreting services, refugees’ rights,* etc. *so that the information will be quickly available and consultable. This guide will be distributed to hospitals, the mental health services,* etc.*”.*

Brussels, mental health care professional for refugees/asylum seekers, Id. 21

Those workers who tried to develop contacts with other organisations sometimes found it difficult to keep up with an ever-changing landscape of short-term, non-governmental projects with limited funding.

“Lack of cooperation and information is really destructive. In fact, we should have a full updated list of NGOs and their projects in the field of social inclusion not only in our district but in all Warsaw, as the Warsaw municipality is one system.”

Warsaw, social care professional for the homeless, Id. 115

Compared to professionals, people with mental disorders in marginalised groups would have even less access to reliable information about services and how to access them.

“In general Travellers have little knowledge of available (mental) health services and would perhaps not know where to turn to.”

Amsterdam, social care professional for travelling communities, Id. 111

Some experts also expressed concern that available health care information does not actually reach people from marginalised groups. They emphasised that points of distribution need to be carefully considered to reach the desired target populations.

“So the first way would be to spread the maximum information possible in the neighborhoods with a larger concentration of immigrant populations, in associations, in churches, in religious gatherings, in the media, stating that services are free of charge, confidential, and whatever is necessary for people to go and use the national health service without fear.”

Lisbon, social care professional for refugees/asylum seekers, Id. 132

The modalities of information delivery and their suitability for particular groups have also been identified factors that need to be improved to ensure good practice.

“Culturally appropriate mental health promotion is what is required… thinking how to provide information for them in an appropriate way, so that it would reach community. For example, taking into account that some Romani dialects are not commonly written and trying to adapt provision of information to this fact by using video or something similar.”

London, social care professional for travelling communities, Id. 176

## Discussion

In interviews with health care, social care and mental health care professionals in 14 European countries, four components characterising good practice in mental health care across six different socially marginalised groups were identified: a) establishing outreach programmes to identify and engage with individuals with mental disorders; b) facilitating access to services that provide different aspects of health care, including mental health care, thus reducing the need for further referrals; c) strengthening the collaboration and co-ordination between different services; and d) disseminating information on services both to the marginalised groups themselves and to health care practitioners in the area.

These components were applicable in different countries and across different marginalised groups.

The study has a number of strengths. A substantial number of experts in 14 countries were interviewed. A consistent methodology for selecting areas and defining marginalised groups was used across all countries. All experts had actual experience providing care to the specific marginalised group they were being interviewed about. The findings reflect experiences gained in countries with very different social care and health care systems and with six groups that represent different types and histories of social marginalisation. The identified good practice components reflect commonalities across countries and groups despite national and group differences, and may therefore be seen as widely applicable.

The above-mentioned strength of the study to draw general conclusions based on material from so many countries and six very different social groups is also a limitation. Such analysis inevitably simplifies and does not reflect the richness of the material. Since the interview was semi-structured and did not explicitly investigate views on pre-defined components of good practice, negative findings (i.e. if experts did not raise a theme) are difficult, if not impossible, to interpret. Interviewees might have held strong views on certain issues but not expressed them in the interview. In fact, none of the 13 themes was raised in all countries and more often than not a theme was mentioned by only one of the two experts in one country. The analysis in this exploratory study reflects only what was put forward as explicit opinions concerning good practice, but did not, for example, go back and test the wider validity of the components with all interviewed experts.

There are further shortcomings of the study. Experts were selected based on local knowledge and experience or research teams, but the recruitment was still opportunistic and may have been inconsistent. The interviews were of different length and detail. Most importantly, the analysis used material that was translated into English, and relevant and specific factors may have been lost in translation. Also, the analysis was mainly conducted by a group based in the United Kingdom with inevitably limited understanding of specific contexts and whose perspective may have been influenced by their own national experience, despite regular cross-checking with all sites. All these aspects can be associated with a possible interpretation bias. Finally, the study having been conducted in highly deprived urban areas, findings cannot necessarily be generalisable to other types of settings.

The identified components are based on a common denominator across countries and groups. They are therefore rather general and reflect widely held views among experts. For some of the groups similar components have been suggested in the literature previously [26-33]. Outreach was described as an important component, which is in line with an increasing consensus in the literature that outreach activities are an essential element of community mental health care for difficult to engage groups [34,35]. These activities are also increasingly recognised form of delivering other health care to individuals from marginalised groups whose needs are not effectively addressed by existing services [36-38]. However, outreach may be particularly significant in mental health care. Experts pointed towards a requirement for close contacts and proactive support whilst at the same time avoiding any form of intrusiveness. Services have to take responsibility for providing health care to vulnerable groups and at the same time respect their autonomy. This ongoing tension between paternalism and respect for autonomy applies to relationships between clinicians and individual patients, and between services and specific communities [39]. Lack of trust in services was a frequent theme. Health care may be resisted in socially marginalised communities if it is perceived as a threat to a group’s autonomy [40]. Respect for the autonomy of marginalised groups may require increasing professionals’ awareness of the particular social issues of those groups among professionals [41] and subsequently adapting service provision to the different lifestyles [42].

Facilitating access may require overcoming institutional inertia and is linked to initiatives of having person centred care instead of institution centred care [43,44]. The requirements may vary by groups and by size of institution. For example, a very small service may be able to establish personal links and trust, but struggle to ensure opening times beyond office hours. Yet, the findings of this study suggest that the major challenge to mental health services is not only ease of access, but to be closely linked to general health care and help patients access all health care and treatments as and when required. A split between mental health and other health care might sometimes simplify the organisation of services, but it is not in the interest of marginalised groups who have difficulties navigating complicated health care systems and whose main and initial focus is often obtaining care for general health problems rather than mental health care.

The coordination of services becomes more difficult the more services there are and can potentially be involved in providing care for marginalised groups. Other European reports on health needs of marginalised groups have described problems in working across traditional areas of responsibility [45,46]. Well resourced health and social care systems tend to have more services [47], and although this may widen the range of available care options for patients, it will also make coordination more complicated. Of all the identified components of good practice, coordination of services appeared to many interviewees the easiest to achieve with limited additional activities and input. At the same time, the isolation of services and their fragmentation have been identified as a problem previously without being overcome. An increasingly diverse landscape of provider organisations might make this even more difficult.

It may seem surprising that simple information on care options and existing services is often not available, yet this finding was frequently reported by the experts from different care domains. Similar to problems with coordination, this issue is linked to working across usual boundaries which is often a necessity in case of marginalised groups. The authorities overseeing and coordinating health and social care services may be seen as the most obvious candidate for providing such information, and new technologies should facilitate the compilation of the required data. More challenging than compiling the data is getting the information to marginalised groups and providing it in a form that is suited to their needs, a finding supported by other European studies [48]. Marginalised groups often do not actively search the internet or other sources for information. Helping them to do so may be one important task for services which provide the first point of contact to these groups.

Implementing the good practice components can be achieved through sufficient funding, appropriate service organisation and training of staff. Establishing outreach programmes and providing and disseminating information require resources. Organisational development may help to reduce administrative barriers and complex referral procedures focusing on outreach and establish good collaboration and co-ordination between services. Training and supervision programmes in both specialised and generic services may enable staff to develop a better understanding of the specific needs of marginalised groups; improve their awareness of the existence of other relevant services in the area and how these other services function; facilitate collaboration, and help develop a respectful, non-intrusive approach.

## Conclusions

Identifying components of good practice that apply to several marginalised groups may prove to be a useful way for guiding the development of services, since care systems struggle to provide very different approaches and pathways for each group. Specific approach of services in specific countries with specific population groups - for example when developing methods for establishing trust in an outreach programme - requires however more detailed evidence on good practice for each situation. Future research should analyse how the general components should be specified and complemented for each group and possibly also for different national and local contexts.

New strategies and policies are being developed to improve mental health care across Europe. As emphasised by the EC Green Paper on mental health, the specific needs of marginalised will have to be addressed in future policies to reduce inequalities in the provision of care and to strengthen social cohesion [49]. The findings of this study suggest that there is a widely shared understanding across different countries on how services should be provided and that policies can be based on at least some international consensus among experts on what might constitute good practice in service organisation and delivery.

## Competing interests

The authors declare that they have no competing interests.

## Authors’ contributions

SP, AM, CS, HB, MB, JMDO, EG, TG, PH, UK, VL, JM, AS, GM and AG all made substantial contributions to the design of the study, data collection, interpretation of the findings and critical revision of drafts. AM and CS further contributed to data analysis. RS contributed to data analysis, interpretation of the findings and drafting of the manuscript for publication. All authors read and approved the final manuscript.

## Pre-publication history

The pre-publication history for this paper can be accessed here:

http://www.biomedcentral.com/1471-2458/12/248/prepub
